# Comparison of Diagnostic Accuracy of Real-Time Elastography and Shear Wave Elastography in Differentiation Malignant From Benign Thyroid Nodules

**DOI:** 10.1097/MD.0000000000002312

**Published:** 2015-12-31

**Authors:** Wuguo Tian, Shuai Hao, Bo Gao, Yan Jiang, Shu Zhang, Lingji Guo, Donglin Luo

**Affiliations:** From the Department of Breast, Thyroid, and Vascular Surgery, Research Institute of Surgery, Daping Hospital, Third Military Medical University, Chongqing, China.

## Abstract

Thyroid nodules are relatively more prevalent in iodine-deficiency area, and the incidence increased sharply in the past decade in these areas. Workup of malignant from benign nodules in clinic was the main problem for managing thyroid nodules.

An overall search for the articles about the diagnostic performance of real-time elastography (RTE) and shear wave elastography (SWE) before April 2015 in the databases of PubMed, Embase, and Google scholar. The pooled sensitivity, specificity, and summary receiver operating characteristic (SROC) curve were obtained from individual studies with a random-effects model. Subgroup and meta-regression analysis were also performed.

Fifty-six studies involved in 2621 malignant nodules and 7380 benign nodules were contained in our meta-analysis. The pooled sensitivity and specificity of RTE was 83.0% and 81.2%, which is higher than SWE (sensitivity: 78.7%, specificity: 80.5%). The areas under the SROC curve of RTE and SWE were 0.885 and 0.842 respectively. RTE had higher diagnostic value for Caucasians than Asians. Stran ratio (SR) assessment had higher diagnostic performance than elasticity score (ES) system. Similarly, it had higher diagnostic value when malignant nodules were more than 50.

In summary, the results revealed that RTE had higher diagnostic performance than SWE in differentiating malignant from benign nodules. However, future international multicenter studies in the region of thyroid risk need to further assess the diagnostic performance of RTE.

## INTRODUCTION

Thyroid nodules are relatively more prevalent in the iodine deficiency area, and the incidence increased sharply in the past decade.^1^ The emerging of thyroid nodule is a consequence of abnormal growth of thyroid cells, including malignant or benign cells within the thyroid gland.^2^ About 10 to 20 million Americans had obvious thyroid nodules clinically and less than 5% of all the thyroid nodules were malignant.^3^ Therefore, differentiating malignant from the benign nodules in clinic was the main problem for managing thyroid nodules.

According to histological examinations, 5% to 10% of the palpable nodules were malignant.^3^ Meanwhile, if the nodules were stiffer than the normal nodules, physical examination can be used to diagnose malignant nodules directly. Ultrasound is also an effective tool for diagnose thyroid nodules, but it has a relatively low accuracy in differentiating malignant from benign nodules.^4^ The sensitivity and specificity of ultrasound for diagnosis malignant nodules were 55% to 95% and 52% to 81%, respectively, according to the sonographic characteristics of malignant nodules such as spot microcalcification, spiculated margins, hypoechogenicity, intranodular vascularity, and blurred boundaries.^3,5,6^ At the present, fine-needle-aspiration (FNA) biopsy is a minimally invasive method for evaluating thyroid nodules. Though it is still one of the most common methods of diagnosing malignant nodules, its sensitivity and specificity fluctuate so widely (54%–90% and 60%–98% respectively).^7^ Second, serious shortcomings such as the discomfort of patients and serious complications are also the deficiencies of the method. Aside from these shortcomings, costs are the main drawback.^7^ Therefore, another noninvasive method to evaluate thyroid nodules is needed.

Elastography, which has emerged as a potential diagnostic method for thyroid nodules in the future, can overcome the limitations of conventional detection technique. Elastography can distinguish malignant from benign nodules according to the elasticity or stiffness of the tissue.^8^ Real-time ultrasound elastography (RTE) and shear wave elastography (SWE) are the two types of elastography. RTE, which was called electronic palpation and first implemented by Ophir et al^9^, is a new technique used to get the tissue stiffness information noninvasively. RTE is based on the mechanism that softer parts of tissues deform more easily than harder parts under compression, and the degree of distortion of a tissue under an external force can be recorded, thus allowing an objective determination of tissue stiffness.^2^ The elasticity score (ES) and strain ratio (SR) assessment were 2 qualitative evaluating scoring systems of RTE, and they revealed tissue stiffness through a second-generation elastographic device.^10^ Different from the technique of RTE, SWE diagnoses thyroid nodules using high-frequency linear probes under the stimulation of acoustic radiation force impulse (ARFI), and it can reveal the elasticity region clearly and analyze the tissue stiffness according to the expression of Young's modulus (kPa).^11,12^ Based on the formula of Young's modulus, tissue elasticity can be measured by the speed of shear wave propagation, and the softer tissue can be differentiated from the stiff tissue taking into account the different propagation speeds of the shear wave. Indeed, with this method the softer tissue is being presented in blue and the stiffer tissue in red. This technique is quantitative, duplicable, and operator-independent.^13^ Up to now, many studies with small samples evaluated the accuracy in differentiating malignant from benign nodules using the 2 techniques and presented promising results. However, owing to the small number of nodules, results of the independent studies had relatively low statistical power. At the same time, few studies compared the diagnostic performance of RTE and SWE in differentiating malignant from benign nodules.

The aim of the present study was to evaluate the diagnostic value of RTE and SWE for distinguishing malignant from benign nodules through performing the overall comprehensive meta-analysis. Meanwhile, we also did the subgroup analysis of RTE by ethnicity, scoring system, ES pattern, and number of malignant nodules.

## METHODS

Ethic approval was not necessary for this meta-analysis.

### Literature Search Strategy

We conducted an overall search for the articles about the diagnostic performance of RTE and SWE before April 2015 in the databases of PubMed, Embase, and Google scholar. Computer search was performed using the Medical Subject Heading (MeSH) and the keywords, such as “thyroid neoplasms” or “thyroid nodule” or “thyroid carcinoma” or “thyroid cancer” or “thyroid tumor” and “diagnosis” or “diagnosis and examinations” or “sensitivity and specificity” or “sensitivity” or “specificity” or “ROC curve” and in combination with the method of diagnosis “elastography” or “real-time ultrasound elastography” or “tissue elasticity imaging” or “elasticity imaging technique” or “shear wave elastography.” Meanwhile, to avoid missing data, we also reviewed the references of the eligible articles manually. All the included articles were all English or Chinese and the literature searching was performed by 2 authors independently.

### Inclusion and Exclusion Criteria

All the articles were first assessed by the title and abstract, and then by the full text. All the articles included in the meta-analysis were all the independent studies of evaluating the diagnostic accuracy of RTE and SWE in differentiating malignant from benign nodules. Meanwhile, articles were included if they meet the following criteria: the independent study subject is in differentiating malignant from benign nodules, all the samples of the control group must be the patients of benign nodules, biopsy, cytology, or pathological result was used as reference standard, it must offer the data of sensitivity and specificity or the ratio of true positive, false positive, true negative, and false negative, the imaging technology is RTE or SWE. We excluded articles if they were not English or Chinese. Disagreements were negotiated by the third authors.

### Data Extraction

Two authors, working independently of each other, extracted data from the full text using a uniform form. Characteristics (such as sample size, number of nodules) were extracted from the full-text articles and assessed by 2 reviewers (2 other persons) independently. Data on publication information (eg, author's first name, year of publication), patient characteristics (country, ethnicity, sample size, number of malignant and benign nodules), type of elastography, scoring system (ES or SR), ES pattern, diagnosis accuracy (sensitivity and specificity). All the disagreements were resolved by a third author.

### Statistical Analysis

Statistical heterogeneity was estimated by the *χ*^2^ test. If *P* < 0.05 indicates the existence of substantial heterogeneity^14,15^ and we calculated the pooled estimates with the random-effects model which involves an assumption that the effects being estimated in the different studies are not identical, but follow some distribution. Otherwise, the fixed-effect model was selected for analysis if we assume that all studies in the meta-analysis share a common effect size. However, the random effects model is not an alternative for the investigation of heterogeneity as a wide range of factors might contribute to the presence of heterogeneity.

The diagnostic value accuracy of the RTE and SWE was estimated by the pooled sensitivity, specificity, area under the curve (AUC), and partial AUC. Summary receiver operating characteristics (SROC) curves were generated to present the test parameter results using the method of inverse variance. Meta-regression was used to measure the potential factors of heterogeneity such as ethnicity, scoring system, ES pattern, and number of malignant nodules. Subgroup analysis was performed to discuss the different diagnostic performance among different subgroups. Publication bias of the included studies was assessed by Deek's funnel plot asymmetry test. Meta-analysis was conducted by the statistical software of R 3.12. It indicated statistical significance if *P* value was less than 0.05.

## RESULTS

### Characteristics of the Included Literature

**FIGURE 1:**
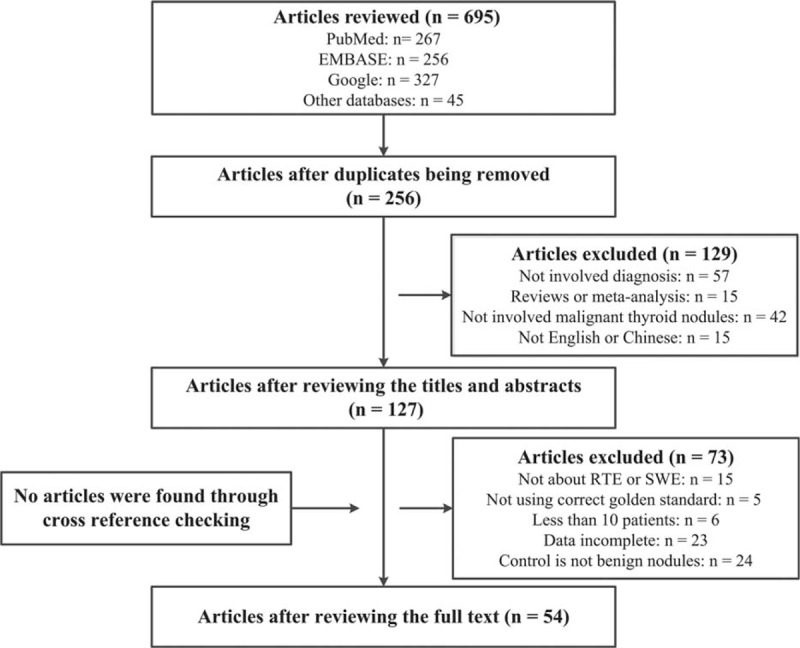
Literature searching flow.

According to the searching strategy, 256 articles were retrieved. Of those, 49 articles were found to fit to our selection criteria and were included in the meta-analysis and the detailed searching flow was presented in Figure 1.^1,7,8,10,11,13,16–59^ The included articles, which were published between 2005 and 2015, evaluated 10,001 thyroid nodules (2621 malignant nodules and 7380 benign nodules). The number of analyzed malignant nodules ranged from 10 to 375 and 30 studies had malignant nodules less than 50. ES (37 studies) and assessment of SR (10 studies) were the 2 qualitative evaluation scoring systems used for RTE. Of all the studies using the ES scoring system, 19 studies used a 1 to 4-point scale. All included studies used the golden standard (Biopsy, cytology, histological findings, pathologic, or pathological result) as a reference standard. The detailed characteristics of the included studies are summarized in Table 1.

**TABLE 1:**
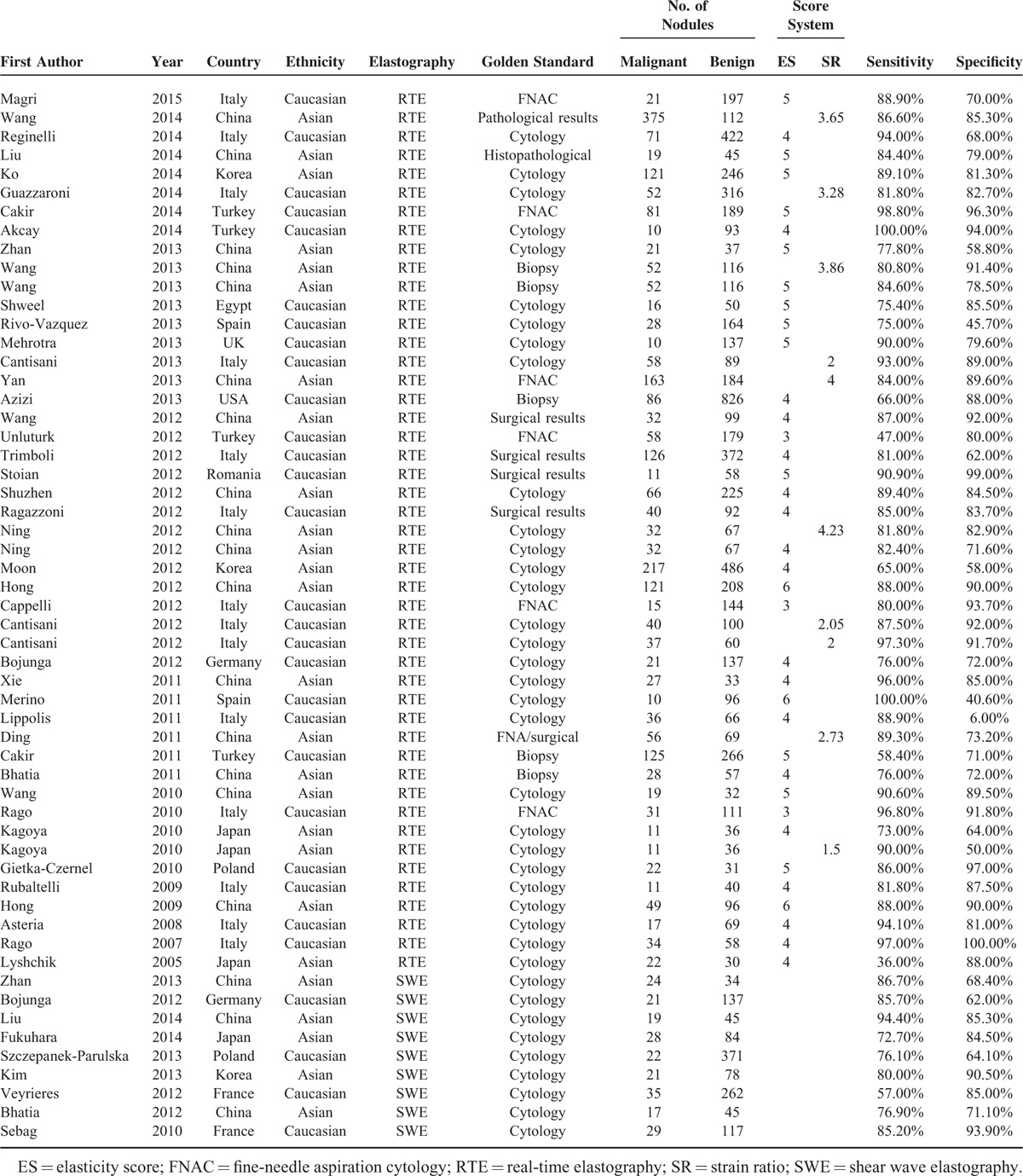
Main Characteristics of the Selected Studies in the Meta-Analysis

### Summary Estimates of Differentiation Between Malignant and Benign Thyroid Nodules

**FIGURE 2:**
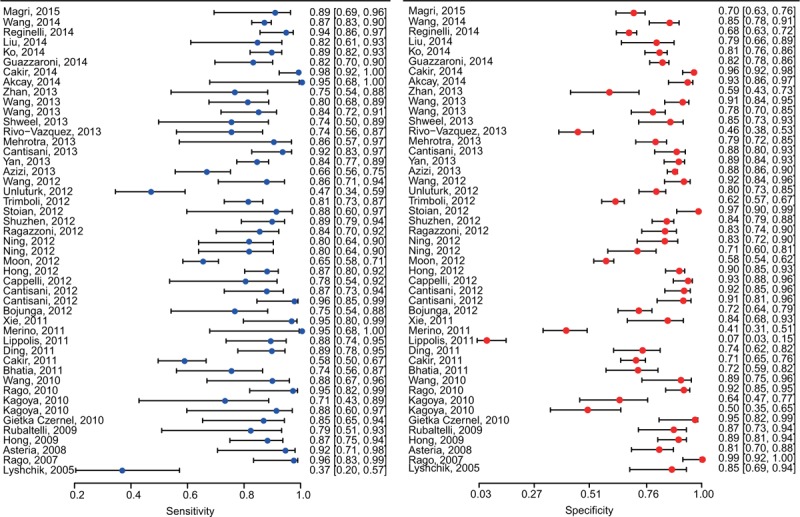
Forest plot for RTE.

**FIGURE 3:**
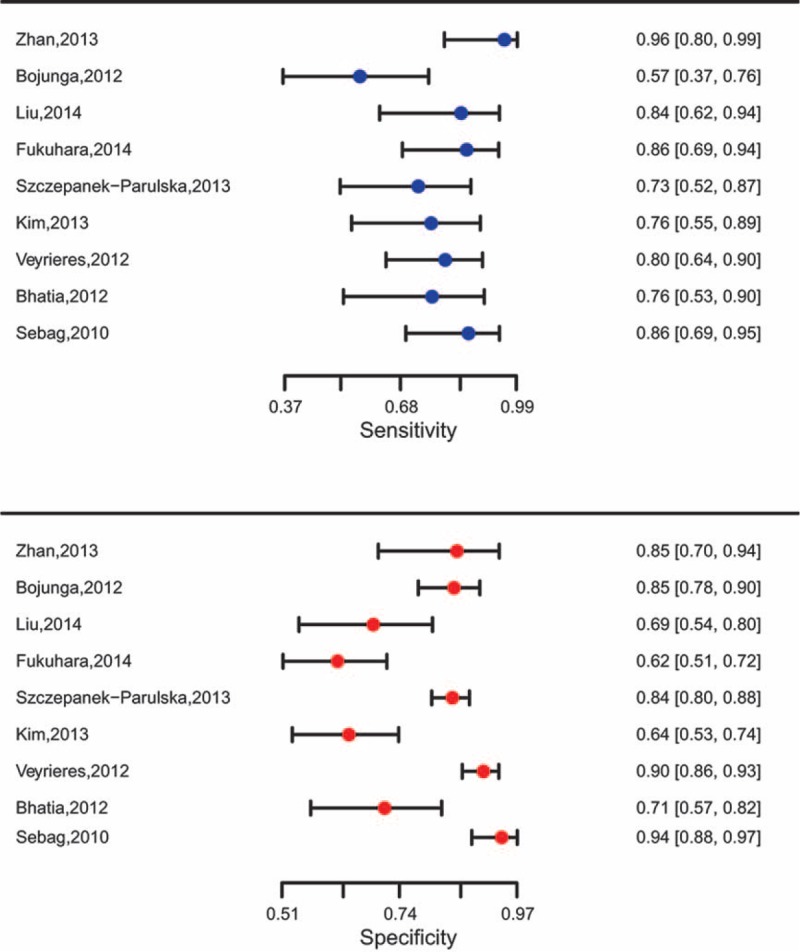
Forest plot for SWE.

**FIGURE 4:**
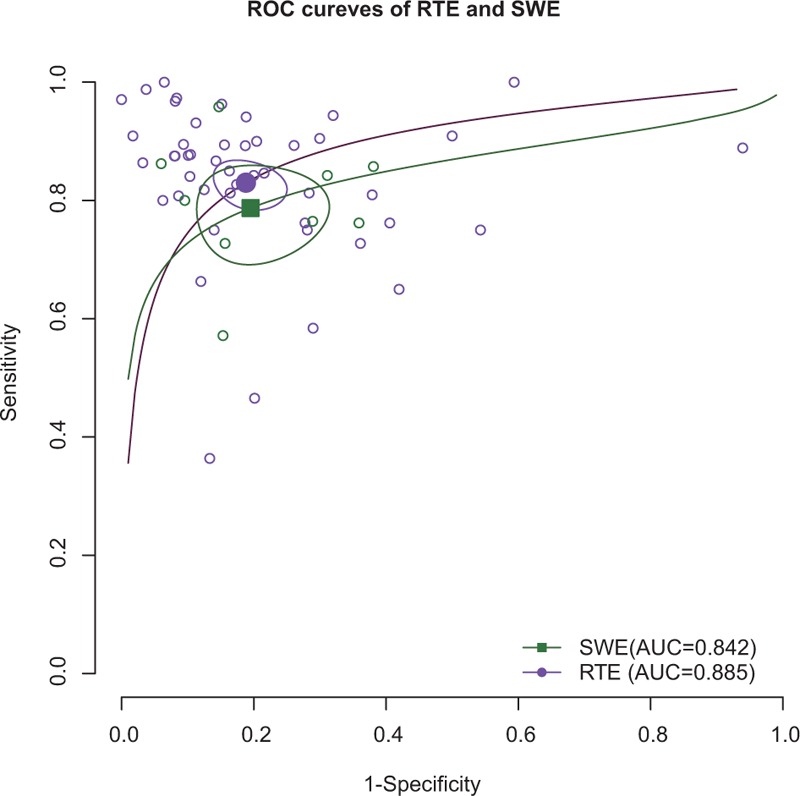
Compare of RTE and SWE using SROC curves.

Forest plots of RTE and SWE on the sensitivity and specificity in diagnosing malignant from benign thyroid nodules are shown in Figures 2 and 3. Random-effects model was used to conduct the pooled sensitivity and specificity of the included studies, and the results are shown in Table 2. The pooled sensitivity and specificity of RTE were 0.830 (95% CI: 0.793–0.861) and 0.812 (95% CI: 0.763–0.852) respectively. Figure 4 shows the result of SROC curve and the summary AUC was 0.885. Meanwhile, we calculated misdiagnosis rate (eg, false-positive) and missed diagnosis rate (eg, false-negative) of RTE were 16.01% and 5.03%. All the results revealed that RTE was efficient in differentiating malignant from benign nodules.

**TABLE 2:**
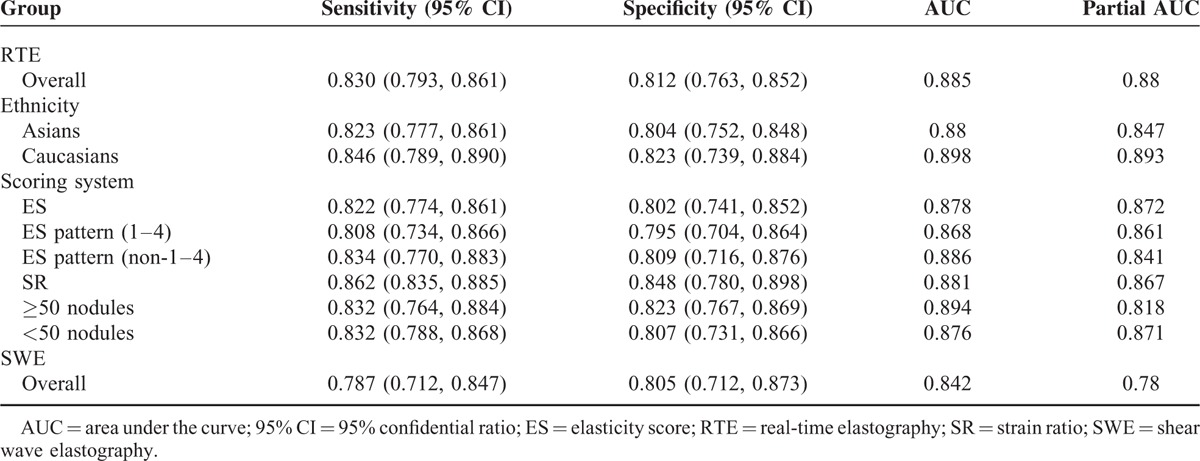
Summary Parameters for Assessment of Elastography (RTE&SWE)

Similarly, SWE also demonstrated a relative accurate diagnosis value in excluding malignant nodules, with the pooled sensitivity of 0.787 (95% CI: 0.712–0.847) and the specificity of 0.805 (95% CI: 0.712–0.873) (Table 2). The AUC of SROC curve was 0.842 which was shown in Figure 4. Misdiagnosis rate (eg, false-positive) and missed diagnosis rate (eg, false-negative) of SWE were 14.61% and 3.10% respectively.

### Subgroup Analysis in Differentiation Between Malignant and Benign Thyroid Nodules

**FIGURE 5:**
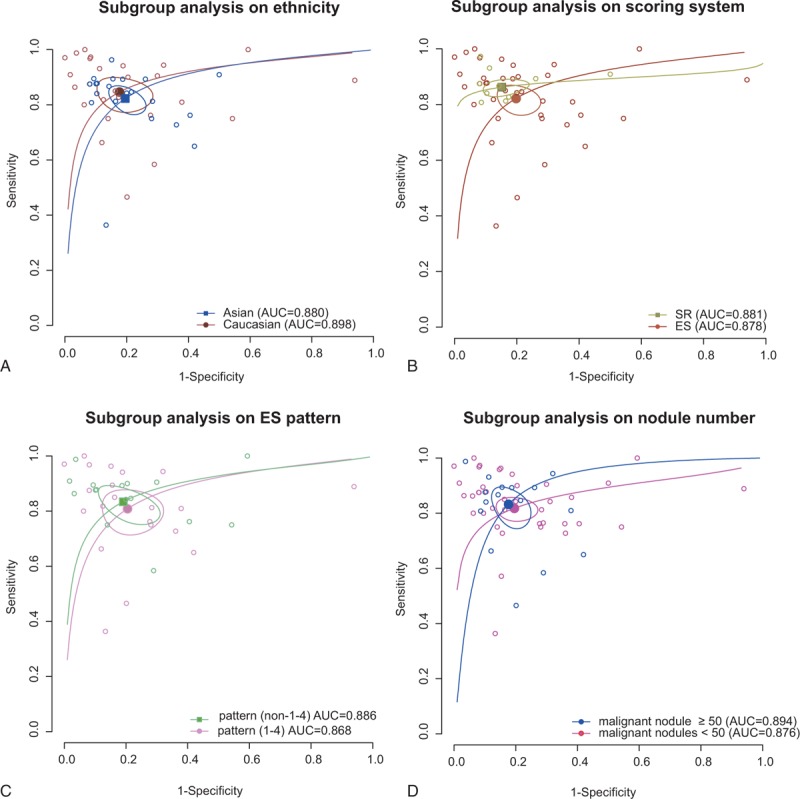
Subgroup analysis for RTE based on ethnicity (A), scoring system (B), ES pattern (C), and malignant nodule number (D).

Subgroup analysis by ethnicity, scoring system, ES pattern, and number of malignant nodules was conducted and the results are shown in Table 2. RTE had higher diagnostic value for Caucasians (sensitivity: 0.846, specificity: 0.823, AUC: 0.898) than Asians (sensitivity: 0.823, specificity: 0.804, AUC: 0.880) (Figure 5A). The result of scoring system revealed that SR assessment was higher than assessment of ES, with sensitivity of 0.862, specificity of 0.848, and AUC of 0.881 (Figure 5B). The results of ES pattern showed that non-1–4 ES pattern was higher than 1–4 ES pattern (Figure 5C). Similarly, it had higher diagnostic value when malignant nodules were more than 50 (Figure 5D).

### Meta-Regression Analysis

Because noticeable heterogeneity was found in the test of sensitivity and specificity (*P* ≤ 2 × 10^–16^) meta-regression analysis was conducted in our study to discuss the potential sources of heterogeneity, containing ethnicity (Asians vs Caucasians), type of elastography (RTE vs SWE), number of malignant nodules (≥50 vs < 50), scoring system (ES vs SR), and ES pattern (1–4 pattern vs non-1–4 pattern). All the analyzed elements could affect the pooled sensitivity and specificity significantly.

### Publication Bias

Deeks’ funnel plot asymmetry test was used to test publication bias. It is conducted using a regression of the diagnostic log odds ratio against 1/sqrt (effective sample size) weighting by effective sample size, with *P* < 0.10 for the slope coefficient suggesting significant asymmetry.^60^ The results revealed that no publication was found, with *P* value of 0.13 for RTE and 0.39 for SWE.

## DISCUSSION

Thyroid nodules occur commonly, and palpable nodules were found in 5% of the population. The number of thyroid nodules increased to 50% after autopsy or sonography.^1^ Therefore, the diagnosis of thyroid nodules becomes the main focus in clinical practice. Conventional sonography can detect thyroid nodules accurately although it has poor diagnostic value in differentiation of malignant from benign nodules, and the main diagnostic method of thyroid nodules was minimally invasive FNA. However, the diagnostic accuracy of FNA was limited by many factors, such as cell samplings, size of nodules, and hemorrhagic cysts.^53^ Differentiation of malignant from benign nodules becomes difficult or even impossible when the FNA samples with adequate cells, especially in the distinction between a benign follicular adenoma and a follicular carcinoma.^61^ Meanwhile, 30% malignant nodules were missed if nodules were too small or patients had a fibrotic thyroid.^62^ In recent years, RTE and SWE were used as the new noninvasive techniques for diagnosis thyroid nodules. The present study aims to evaluate the efficiency of RTE and SWE in the diagnosis of thyroid nodules. The results of our meta-analysis, which included 54 studies and 2621 malignant nodules, revealed that RTE had higher pooled sensitivity and specificity than SWE for differentiating malignant and benign nodules. The AUC of SROC curve revealed that RTE had a relatively higher diagnostic accuracy than SWE. According to the results, RTE can be used as an effective diagnostic tool for differentiation malignant nodules in clinical practice.

Elastography was widely applied to the diagnosis of prostate cancer, breast cancer, and lymph node metastasis.^63–65^ RTE, which is a semiquantitative elastography, plays an important role in differentiation of malignant from benign nodules. Cappelli et al^38^ found that RTE had high accuracy even in differentiation small thyroid nodules (maximum diameter was 3–10 mm) compared with the conventional techniques, with sensitivity of 0.91 and specificity of 0.89. Compared with FNA and conventional sonography, RTE is more suitable for differentiation malignant from benign thyroid nodules in the present study^48,50,62^ and the results were also demonstrated by many independent studies, though Alexander^66^ found that RTE was limited in assessing multinodular goiters. SWE, one of the most common types of elastography, was also used by several authors in the field of clinical diagnosis.^67–69^ It is a technique with a highly reproducible procedure and can clearly distinguish large or small malignant nodules, even in the patients with multinodular goiters.^69^ However, Sebag et al^59^ found that SWE had a high false-positive rate for differentiation malignant nodules with macrocalcifications. Overall, RTE had a higher diagnostic value than SWE in differentiation between malignant and benign nodules.

ES evaluation and assessment of SR were the 2 different evaluation ways of RTE in distinguishing malignant nodules. ES system is a qualitative assessment analysis, which relies on observer independently and is widely acknowledged in the field of clinical practice.^59^ SR assessment is a semiquantitative analysis and the result is affected by examining doctor skills or experience.^59^ The ES and SR were all higher in malignant than benign nodules. Therefore, both of them were useful indexes in differentiation of malignant from benign nodules. Our results of subgroup analysis showed that assessment of SR had a higher sensitivity and specificity than ES system in distinguishing malignant from benign nodules, and the results were in accordance with the results of previous studies.^22,33^ The potential reason for the difference of ES and SR in diagnostic performance may be caused by the decrease of pressure with increasing depth for the error of observers.^33^ Furthermore, subgroup analysis results of ethnicity, ES pattern, and number of malignant nodules were consistent with the meta-analysis results of Sun et al.

Heterogeneity existed in sensitivity and specificity of studies included in the meta-analysis. The reason may be caused by the bias of spectrum composition in different studies. Meanwhile, another explanation appeared to be the low statistical power, which could be caused by the relatively small sample size of independent studies, especially the studies regarding malignant nodules of SWE. Furthermore, other factors to explain the large heterogeneity observed in the results reported in different studies such as the different equipment used (eg, sonographic equipment, RTE software and hardware capabilities) and observers’ experience may also play an important role in heterogeneity among studies.

Owing to the fact that 5 meta-analyses published previously missed several studies, our meta-analysis is the most comprehensive meta-analysis to assess the performance of diagnosis in differentiation malignant from benign nodules using RTE and SWE. However, it had several limitations. First, the cutoff values of RTE ranged from 1.50 to 4.23 in different studies. Furthermore, it cannot do subgroup analysis because of limited number of studies. Second, the results of diagnostic performance of RTE and SWE may be affected by the complicated neck structure such as carotid artery, trachea. Finally, there are a small number of studies concerning the second elastography method (SWE). The main cause is that RTE exist on the market more than 15 years, whereas SWE is a more recent development and therefore relevant studies can find only in recent bibliography. This issue may have influenced the results. Therefore, further investigations and confirmations are needed so as to provide more evidence and clinical guidance on which is the most reliable method for the differential diagnosis of thyroid nodules.

In summary, our meta-analysis revealed that RTE had higher diagnostic performance than SWE in differentiation of malignant from benign nodules. Future international multicenter studies in the investigational area of thyroid nodules need to further assess the diagnostic performance of RTE and SWE.
