# Comparison of Diagnostic Accuracy of Real-Time Elastography and Shear Wave Elastography in Differentiation Malignant From Benign Thyroid Nodules: Erratum

**DOI:** 10.1097/01.md.0000481322.75086.b6

**Published:** 2016-03-03

In the article “Comparison of Diagnostic Accuracy of Real-Time Elastography and Shear Wave Elastography in Differentiation Malignant From Benign Thyroid Nodules”,^1^ which appeared in Volume 94, Issue 52 of *Medicine*, Dr. Lingji Guo's name was originally spelled incorrectly. The article has since been corrected online.
